# Linoleic acid metabolite 13-Hydroxyoctadecadienoic acid as a biphasic ferroptosis modulator in granulosa cells: multi-omics analysis of ovine atretic follicles

**DOI:** 10.3389/fcell.2025.1610621

**Published:** 2025-05-30

**Authors:** Yukun Song, Erhan Hai, Lixia He, Ning Zhang, Nan Zhang, Junlan Wang, Yupeng Sun, Dengke Zeng, Jiaxin Zhang

**Affiliations:** ^1^ Inner Mongolia Key Laboratory of Sheep and Goat Genetics Breeding and Reproduction, College of Animal Science, Inner Mongolia Agricultural University, Hohhot, Inner Mongolia, China; ^2^ Key Laboratory of Mutton Sheep and Goat Genetics and Breeding, Ministry of Agriculture and Rural Affairs, Hohhot, Inner Mongolia, China

**Keywords:** ovine atretic follicle, ferroptosis, 13-hydroxyoctadecadienoic acid, lipid metabolism, transcriptome and metabolome

## Abstract

**Introduction:**

13-Hydroxyoctadecadienoic acid (13(S)-HODE) is a bioactive lipid derived from linoleic acid, it plays prominent roles in cellular processes such as lipid metabolism, oxidative stress, and apoptosis. Follicular atresia is a complex physiological process involving multiple forms of cell death. Ferroptosis, an iron-dependent form of programmed cell death, has been less studied in the context of follicular atresia.

**Methods:**

To investigate the association between ovine follicular atresia and ferroptosis, we performed transcriptomic and metabolomic analyses of healthy and atretic sheep follicles. Notably, sheep follicular granulosa cells were treated with different doses of 13(S)-HODE. Cell viability, lipid peroxidation levels, ferroptosis-related markers, and ferroptosis-related genes were measured.

**Results:**

The metabolomic analysis identified 87 and 48 differentially expressed metabolites in healthy and atretic follicles, respectively. Functional enrichment of atretic follicle fluid highlighted pathways related to linoleic acid and purine metabolism. Transcriptomic analysis revealed 250 highly expressed genes in ovarian granulosa cells of atretic follicles. Enrichment analysis indicated that these differentially expressed genes were associated with fatty acid metabolism and ferroptosis. Integration of multi-omics data demonstrated the occurrence of ferroptosis in atretic follicles, where 13(S)-HODE drives granulosa cell ferroptosis via the linoleic acid metabolism pathway; this effect was not dose-dependent. Mechanistic studies showed that low-dose 13(S)-HODE counteracts ferroptosis by promoting glutathione peroxidase 4-mediated lipid peroxidation reduction and increasing glutathione levels.

**Discussion:**

In contrast, high-dose 13(S)-HODE induces labile iron accumulation through activation of transferrin receptor and ferritin heavy chain 1, enhancing ferroptosis sensitivity in granulosa cells. These findings provide insights into the molecular mechanisms regulating follicle development and offer potential therapeutic targets for enhanced follicular development and improved reproductive outcomes.

## 1 Introduction

The ovary is an essential reproductive organ responsible for oocyte production, sex hormone secretion, and estrous cycle maintenance. More than 99% of ovarian follicles undergo atretic degeneration before ovulation (Shen et al., 2017). Damage to granulosa cells can result in follicular atresia and oocyte degeneration (Shen et al., 2021). Previous studies have demonstrated that granulosa cells in atretic follicles exhibit alterations in apoptosis, autophagy, mitochondrial function, and inflammatory responses, contributing to an unfavorable microenvironment for follicle development and oocyte maturation (Zhang et al., 2019; Zhou et al., 2019; Liu et al., 2023). There is emerging evidence of a relationship between ferroptosis and granulosa cells, indicating that ferroptosis-related genes may regulate granulosa cell proliferation and ovarian reserve function (Chen et al., 2023; Zhang S. et al., 2023). Follicular fluid plays a crucial role in the follicular microenvironment by supporting granulosa cell proliferation and oocyte maturation while reflecting metabolic processes during follicle development (Ban et al., 2021). Analyses of follicular fluid metabolites could provide valuable information regarding the metabolic status of follicles and facilitate assessments of follicular development. Recent research has characterized the metabolic signatures of human follicular fluid across various follicular stages, providing insights into metabolic changes (Yang et al., 2020; Yang et al., 2022). Linoleic acid, an essential n-6 polyunsaturated fatty acid (PUFA), it is exclusively obtained through diet and serves as a precursor for the synthesis of inflammatory substances (Poli et al., 2023). Other clinical analyses have found a significant increase in linoleic acid concentration in patients with polycystic ovary syndrome (PCOS), and have found that reactive oxygen species-related oxidative stress occur downstream of LA (Zhang and Wu, 2024). Tan et al. (2024) found that changes in the oxidative stress and lipid metabolism pathways play important roles in PCOS pathogenesis. Roura et al. (2018) found that high concentrations of linoleic acid in follicular fluid impairs oocyte membranes in goats. Similarly, Amini et al. (2016) also found that high concentrations (200 μM/mL) of linoleic acid significantly reduced the *in vitro* maturation and embryonic development ability of sheep. 13-Hydroxyoctadecadienoic acid (13(S)-HODE) is a bioactive lipid derived from linoleic acid through the action of 15-lipoxygenase (Vangaveti et al., 2010); it plays prominent roles in cellular processes such as lipid metabolism, inflammation, oxidative stress, and apoptosis (Cabral et al., 2014). Research has found that 13-HODE induces mitochondrial dysfunction and airway epithelial cell damage through ROS mediated oxidative stress (Mabalirajan et al., 2013). However, whether linoleic acid-induced oxidative stress is caused by its metabolite 13(S)-HODE plays a role in follicular atresia in sheep. We reasonably hypothesized that excessive linoleic acid is metabolized by lipoxygenase to generate high levels of 13(S)-HODE, which leads to abnormal accumulation of reactive oxygen species in the follicular microenvironment, which in turn triggers oxidative damage in granulosa cells, ultimately accelerate the process of follicular atresia.

Ferroptosis is a recently identified iron-dependent form of programmed cell death characterized by lethal levels of iron-mediated lipid peroxidation (Liu et al., 2020). Biochemically, it is distinguished by increased concentrations of iron ions, excessive production of reactive oxygen species (ROS), reduced activity of glutathione peroxidase 4 (GPX4), and inadequate clearance of lipid metabolites, resulting in cellular and tissue damage (Yu et al., 2021). In an environment exhibiting uncontrolled lipid peroxidation and apoptosis, disruption of the antioxidant balance may trigger ferroptosis in granulosa cells, ultimately leading to follicular atresia. There is evidence that GPX4 prevents ferroptosis by eliminating intracellular peroxides and preserving cell viability (Xie et al., 2023). Iron, an essential micronutrient, is a core component of biological processes such as oxygen transport, DNA replication, and redox reactions (Stockwell et al., 2017). Ferritin, the primary iron storage protein, consists of 24 subunits formed by heavy and light ferritin chains. Reduced expression of ferritin heavy chain 1 (FTH1) increases ferritin degradation and promotes ferroptosis (Hou et al., 2016). The transferrin receptor (TFRC) plays a key role in regulating receptor-mediated iron uptake and is indispensable for ferroptosis (Zhou X. et al., 2024). After TFRC binds to transferrin-carrying Fe^3+^, the transferrin-TFRC complex facilitates iron uptake via endocytosis (Kawabata, 2019). Recent evidence suggests that ferroptosis is associated with the oocyte quality (Song et al., 2025) and freezing-induced sperm damage (Hai et al., 2025) in ruminants. A key pathway through which 13(S)-HODE exerts its effects involves the regulation of GPX4. Recent studies have highlighted this regulatory relationship, suggesting a direct link between 13(S)-HODE and the control of ferroptosis (Corteselli et al., 2019; Schwarz et al., 2023). Therefore, revealing the link between 13(S)-HODE-induced alterations in reactive oxygen species and ferroptosis is essential for alleviating follicular atresia.

Untargeted metabolomics, which encompasses a wide range of metabolites, offers advantages in identifying and relatively quantifying metabolites. This approach helps reveal important metabolic signatures and novel biomarkers, yielding insights concerning mechanisms that underlie physiological processes in atretic follicles. In this study, we analyzed abnormal changes in follicular fluid metabolites to identify multiple metabolites and key pathways involved in follicular atresia. Subsequently, we used RNA-seq data to investigate the regulatory mechanisms of granulosa cell transcription factors and key ferroptosis-related genes in follicular atresia. Multi-omics analysis demonstrated the potential roles of lipid metabolism in regulating granulosa cell ferroptosis. 13(S)-HODE was identified as a key modulator of granulosa cell ferroptosis and follicular atresia. Our findings indicated that 13(S)-HODE enhances granulosa cell resistance to ferroptosis by modulating lipid peroxidation and ROS levels. These results contribute to a deeper understanding of the mechanisms governing ferroptosis during follicular development and offer potential biomarkers for enhanced follicular development and improved oocyte health.

## 2 Materials and methods

### 2.1 Morphological classification of follicles and the collection of follicular fluid and granulosa cells

12 healthy adult Chinese Hu sheep (1.5–2.5-year-old), procured in April 2023 from the Inner Mongolia JinLai Animal Husbandry Technology Co., Ltd. (Hohhot, Inner Mongolia), were received the same hormonal treatment, which involved receiving intravaginal progesterone device (CIDR) for 6 days and treated with 360 IU FSH (Sansheng, Ningbo, China) after normal ovulation. These sheep were raised under standard conditions and were given free access to food and water. Based on previous studies (Cheng et al., 2021), antral follicles were classified as healthy or atretic using follicle morphology and cumulus-oocyte complex status. Follicles were first classified according to their appearance, including characteristics such as color, capillarity, and transparency. The classification was further refined using the morphological features of cumulus-oocyte complexes, such as ooplasm quality, cumulus cell quantity and distribution, and follicular fluid turbidity. For this study, follicular fluid and granulosa cells were collected from ovarian tissues as previously described (Song et al., 2024). Briefly, follicular contents were aspirated from antral follicles (3–6 mm) using 29-G needles, and cumulus-oocyte complexes or naked oocytes were isolated under a stereo microscope (SMZ-645; Nikon, Tokyo, Japan). Follicular fluid samples were centrifuged at 1500 *g* for 5 min at 4°C; the supernatant was collected and stored at −80°C for further analysis. Granulosa cells in the precipitate were washed 2–3 times with phosphate-buffered saline, mixed with 500 μL of TRIzol reagent (Ambion, Austin, TX, United States), and stored at −80°C for RNA extraction.

### 2.2 Metabolite extraction and liquid chromatography-tandem mass spectrometry (LC-MS/MS) analysis

Twelve follicular fluid samples from healthy and atretic follicles were used for metabolite extraction. Briefly, 200 μL of follicular fluid was mixed with 900 μL of precooled methanol. The mixture was vortexed and stored at −20°C for 30 min. After centrifugation at 12,000 rpm for 15 min at 4°C, the supernatants were collected for LC-MS/MS analysis. Quality control samples were prepared by combining 10 μL of supernatant from each sample to evaluate the stability of the LC-MS analysis. All chromatographic separations were performed using the UltiMate 3000 UPLC System (Thermo Fisher Scientific, Bremen, Germany) with reversed-phase separation on an ACQUITY UPLC T3 column (100 mm × 2.1 mm, 1.8 μm, Waters, Milford, United States) at a column temperature of 35°C. The mobile phase consisted of solvent A (water) and solvent B (acetonitrile). The flow rate was 0.4 mL/min with an injection volume of 4 μL. The gradient elution conditions were as follows: 0–0.8 min, 2% B; 0.8–2.8 min, 2%–70% B; 2.8–5.6 min, 70%–90% B; 5.6–6.4 min, 90%–100% B; 6.4–8.0 min, 100% B; 8.0–8.1 min, 100%–2% B; 8.1–10 min, 2% B. Eluted metabolites were detected using a high-resolution tandem mass spectrometer Q-Exactive (Thermo Scientific).

### 2.3 Data preprocessing and metabolite–metabolite network analysis

LC-MS/MS raw data were converted into mzXML format using MSConvert software (ProteoWizard, Palo Alto, CA, United States). Quality control, ion annotation, and metabolite identification were performed using the XCMS, CAMERA, and metaX toolboxes implemented in R software (R Foundation for Statistical Computing, Vienna, Austria). Multivariate statistical analysis was conducted using unsupervised principal component analysis (PCA) and supervised partial least squares discriminant analysis (PLS-DA). Additionally, a heatmap was created using the pheatmap package (v3.6.0) in R software. Metabolites with variable importance in projection (VIP) values greater than 1 and *p*-values <0.05 were regarded as differentially expressed metabolites (DEMs). Metabolite annotation was performed using publicly available databases, including the Human Metabolome Database (HMDB) and the Kyoto Encyclopedia of Genes and Genomes (KEGG). Pathway and network analyses of DEMs were conducted using the pathway analysis and network analysis modules of MetaboAnalyst (v5.0).

### 2.4 Processing and analysis of RNA-seq data

RNA extraction and cDNA library construction were performed using previously described methods, in accordance with manufacturer instructions (Li et al., 2021). Total RNA was extracted from granulosa cells of follicles with different statuses (three independent biological replicates per group) using TRIzol reagent. After RNA purity confirmation and rRNA removal, cDNA libraries were constructed using SuperScript™ II Reverse Transcriptase (Cat#18064014; Invitrogen, Carlsbad, CA, United States). Paired-end 150-bp reads were generated on an Illumina NovaSeq™ 6000 platform (LC-Bio Technology Co., Ltd., Hangzhou, China). High-quality clean reads were obtained using Cutadapt (v1.9) and FastQC (v0.11.9) by removing adaptor sequences, reads containing >10% unknown nucleotides (N), and low-quality bases. Clean reads from each sample were aligned to the ovine genome sequence (Ovisaries.Oarv3.1) using HISAT2 (v2.0.4). Alignment results were sorted, analyzed, and indexed with SAMtools. Quantitative analyses of mapped reads were performed using StringTie (v2.1.6) and Ballgown; mRNA abundance was calculated as fragments per kilobase of transcript per million mapped reads (FPKM).

### 2.5 Differentially expressed gene (DEG) identification and Functional annotation analysis

To assess sample reproducibility, unsupervised PCA and Pearson correlation analysis were performed via the ggplot2 (v3.3.3) and CorrPlot (v0.92) packages in R software (v4.0.2), respectively. DEGs between granulosa cells from healthy follicles and those from atretic follicles were identified using | log_2_(fold change) | > 1 and false discovery rate <0.05 with the R package DESeq2 (v1.16.1). Gene Ontology enrichment for biological processes was conducted using the clusterProfiler package (v3.14.3) in R software. KEGG term enrichment analysis was performed using the KEGG Orthology-Based Annotation System (KOBAS-i, v3.0, http://bioinfo.org/kobas) with default parameters. Terms with *p*-values <0.05, adjusted by the Benjamini–Hochberg method, were considered significantly enriched. Protein-protein interaction networks were constructed using the STRING online platform (https://string-db.org/) and imported into Cytoscape (v3.9.0; Cytoscape Consortium, San Diego, CA, United States). Key hub modules within the networks were identified via the CytoHubba and Molecular Complex Detection plugins with default settings.

### 2.6 Integrative analysis of the ferroptosis-related network

An integrative analysis of transcriptomic and metabolomic data was conducted to map ferroptosis-related DEGs and DEMs onto the KEGG database. This process identified common pathways and clarified the key signal transduction pathways regulating ferroptosis in granulosa cells. The MetScape (v3.1.3) plugin for Cytoscape software was applied to analyze the metabolic signatures and transcriptional profiles and then generate pathway maps.

### 2.7 Granulosa cell culture and treatment

Ovarian granulosa cells were isolated from the healthy antral follicles (2–6 mm) of sheep by aspiration with a 29-G needle; they were cultured at a density of 1 × 10^5^ cells/mL in Dulbecco’s Modified Eagle’s Medium/F12 medium (DMEM/F12 medium; Cat#10565018, Life Technologies Corp., Carlsbad, CA, United States) supplemented with 10% fetal bovine serum (Cat#FSP500, ExCell Bio, Shanghai, China) and 1% penicillin–streptomycin solution (Cat#15140122, Life Technologies Corp.). Cultures were maintained at 37°C in a humidified atmosphere with 5% CO_2_. 13(S)-HODE (Cat#GC19463, GLPBIO, Montclair, CA, United States) was dissolved in dimethyl sulfoxide (Cat#D8418, Sigma–Aldrich Chemie GmbH, Steinheim, Germany), ensuring a final dimethyl sulfoxide concentration below 0.1%, then diluted with DMEM/F12 medium to the desired final concentrations. Granulosa cells were plated in six-well plates and allowed to adhere for 12 h, then treated with various concentrations of 13(S)-HODE (25 nM, 100 nM, 250 nM, 500 nM, 1 µM) for 24 h to explore its effects on ferroptosis.

### 2.8 Measurement of cell viability and cell death

To measure cell viability, 1 × 10^4^ cells in 100 µL of medium were seeded in 96-well plates and allowed to adhere overnight. The cells were then treated with the indicated concentrations of 13(S)-HODE for 24 h. Cell viability was assessed by addition of cell counting kit (CCK)-8 reagent (10 μL; Cat#C0037, Beyotime Biotechnology, Shanghai, China) to each well and incubation for 2 h. Absorbance at 450 nm was measured using a microplate reader (Bio-Rad, San Diego, CA, United States). A calcein-acetoxymethyl (AM)/propidium iodide (PI) staining kit (Cat#C2015L, Beyotime Biotechnology) was used to assess live and dead cells. Cells were incubated with 2 μM PI and 5 μM calcein-AM at 37°C in the dark for 30 min. Subsequently, nuclei were stained with Hoechst 33342 staining solution (Cat#C1027, Beyotime Biotechnology). Green, red, and blue fluorescence signals were observed under an Eclipse Ti2-LAPP inverted fluorescence microscope (Nikon, Tokyo, Japan).

### 2.9 Western blot analysis

Granulosa cells were lysed with radioimmunoprecipitation assay lysis buffer (Cat#PR20035, Proteintech, Chicago, IL, United States) supplemented with a protease and phosphatase inhibitor cocktail (Cat#PR20038, Proteintech) to extract proteins. Lysates were clarified by centrifugation at 12,000 rpm for 15 min at 4°C. Clarified lysates were mixed with 5× sodium dodecyl sulfate (SDS) loading buffer (1:5, v/v; Cat#LT101S, Epizyme, Shanghai, China) and incubated at 98°C for 10 min. Protein concentrations were determined using the Omni-Easy Instant BCA Protein Assay Kit (Cat#ZJ102, Epizyme). Protein lysates were separated by SDS–polyacrylamide gel electrophoresis (Cat#P0012A, Beyotime Biotechnology) and wet-transferred to a polyvinylidene difluoride membrane (Millipore, Bedford, MA, United States). Membranes were blocked with Tris-buffered saline plus Tween (Cat#ST671, Beyotime Biotechnology) containing 5% skimmed milk (Cat#PS112L, Epizyme) for 1 h, then incubated overnight at 4°C with the following primary antibodies: GPX4 (1:1000, Cat#30388-1-AP, Proteintech), acyl-CoA synthetase long-chain family 4 (ACSL4; 1:5000, Cat#22401-1-AP, Proteintech), FTH1 (1:1000, Cat#CY5648, Abways, Shanghai, China), Beta Actin (β-actin; Rabbit Polyclonal, 1:4000, Cat#20536-1-AP,Proteintech), and glyceraldehyde-3-phosphate dehydrogenase (GAPDH; rabbit polyclonal, 1:1500, Cat#AF7021, Affinity Biosciences, Queensland, Australia). After membranes had been washed three times with Tris-buffered saline plus Tween, they were incubated with goat anti-rabbit IgG secondary antibody (1:20000, Cat#bs-40295G-IRDye8, Bioss, Beijing, China) or goat anti-mouse IgG secondary antibody (1:20000, Cat#bs-40296G-IRDye8, Bioss) for 40 min at room temperature. Membranes were then washed with phosphate-buffered saline, and fluorescence bands in the 700 nm or 800 nm channels were scanned using the Odyssey CLx Infrared Imaging System (LI-COR Biosciences, Inc., Lincoln, NE, United States). Individual bands were imaged in single channels using ImageJ software (National Institutes of Health, Bethesda, MD, United States).

### 2.10 Flow cytometry analyses of cultured granulosa cells

For intracellular staining, granulosa cells were subjected to fixation and permeabilization using the Intracellular Fixation & Permeabilization Buffer Set (Cat#88-8824-00, eBioscience, Altrincham, United Kingdom), in accordance with the manufacturer’s instructions. Briefly, viable cells were labeled with Fixable Viability Dye eFluor 780 (Cat#65-0865-14, eBioscience) before fixation. Cells were then washed and fixed in the dark at room temperature for 30 min, followed by incubation with permeabilization buffer for 30 min in the dark. Blocking was performed with 5% fetal bovine serum for 60 min. Subsequently, cells were incubated with the following intracellular antibodies: GPX4 (1:50, Cat#30388-1-AP, Proteintech), ACSL4 (1:100, Cat#22401-1-AP, Proteintech), FTH1 (1:100, Cat#CY5648, Abways), solute carrier family 7 member 11 (SLC7A11; 1:100, Cat#26864-1-AP, Proteintech), transferrin (1:100, Cat#17435-1-AP, Proteintech), and TFRC (1:100, Cat#65289-1-Ig, Proteintech). Antibodies were diluted in permeabilization buffer, and cells were incubated for 60 min at room temperature. After incubation, cells were washed and stained with CoraLite® Plus 488-Goat Anti-Rabbit Secondary Antibody (H + L) (1:500, Cat#RGAR002, Proteintech) for 30 min. Flow cytometry analysis was performed using a CytoFLEX LX flow cytometer (Beckman Coulter, Brea, CA, United States), and data were analyzed with FlowJo software (v10, FlowJo LLC, Ashland, OR, United States).

### 2.11 ROS detection, lipid peroxidation, GSH detection, and cellular Fe^2+^ assay

The level of ROS generation was measured using a ROS Assay Kit (dichlorodihydrofluorescein diacetate, Cat#S0033M, Beyotime Biotechnology) and a Dihydroethidium Assay Kit (Cat# S0064S, Beyotime). Lipid peroxidation levels were assessed via BODIPY 581/591 C11 (Cat#D3861, Invitrogen) staining. GSH levels were detected using monochlorobimane (Cat#HY-101899, MedChemExpress, Monmouth Junction, NJ, United States). Granulosa cells were seeded in six-well plates and treated with 13(S)-HODE for 24 h. After treatment, cells were washed twice with serum-free medium and incubated in medium containing 10 μM dichlorodihydrofluorescein diacetate, 2 μM BODIPY 581/591 C11, or 20 μM monochlorobimane; incubation was performed at 37°C in the dark for 30 min. Cells were then washed three times with serum-free medium. Fluorescence intensity was measured using either a flow cytometer or a confocal microscopy, with specific excitation (Ex) and emission (Em) settings. For iron staining, cells were plated in 35-mm glass-bottom dishes. After treatment, cells were washed three times with Hanks’ Balanced Salt Solution and labeled using 1 μM FerroOrange (Cat#F374, Dojindo, Tokyo, Japan) at 37°C for 30 min. Visualization was performed with a confocal microscope (FV1000, Olympus, Tokyo, Japan). To quantify Fe^2+^ levels, cells were seeded in six-well plates. After treatment, cells were resuspended in FerroOrange and incubated for 30 min at 37°C. Fe^2+^ levels were then analyzed by flow cytometry or confocal microscopy.

### 2.12 RNA extraction and reverse transcription quantitative polymerase chain reaction (RT-qPCR) analysis

Total RNA was extracted using the TransZol Up Plus RNA Kit (Cat#ER501-01, TransGen Biotech, Beijing, China). Reverse transcription was performed with the PrimeScript RT reagent kit (Cat#RR047A, Takara, Shiga, Japan). Quantitative real-time PCR (qRT-PCR) was conducted using TB Green® Premix Ex Taq™ II (Tli RNaseH Plus) (Cat#RR820L, Takara) in a StepOnePlus™ Real-Time PCR System (Thermo Fisher Scientific). Gene expression levels were normalized to *Ovis aries* beta-actin (β-actin) mRNA levels, and relative RNA quantification was determined using the comparative cycle threshold (2^−ΔΔCT^) method. Primers were designed using Primer Premier 5 software (Premier Biosoft, San Francisco, CA, United States), and the sequences of gene-specific primers are provided in Supplementary Table S1.

### 2.13 Data analysis

Statistical analyses were performed using GraphPad Prism 10 software (GraphPad, La Jolla, CA, United States). Comparisons between two groups were conducted using Student’s t-test, whereas multiple group comparisons were performed using one-way analysis of variance. Quantitative data are presented as mean ± standard error of the mean. The threshold for statistical significance was regarded as *p* < 0.05.

## 3 Results

### 3.1 Overview of metabolomic profiling

To investigate the effects of follicular atresia on key metabolites and biological markers, we performed non-targeted metabolomic profiling of follicular fluid from the healthy follicular fluid (HFF) and atretic follicular fluid (AFF) groups. The original dataset consisted of 12 experimental samples and eight quality control samples. We conducted a series of data preparation steps and quality control evaluations. The total ion chromatogram results demonstrated consistent retention times and peak intensities, indicating stable signals when the same sample was analyzed at different times (Supplementary Figure S1A). Additionally, Pearson correlation coefficients confirmed high repeatability within groups, ensuring reliable identification of DEMs (Supplementary Figure S1B). After data preprocessing, 284 and 283 metabolites were identified in positive and negative ion modes, respectively (Supplementary Table S2). PCA and PLS-DA were applied to metabolomic data from HFF and AFF, revealing satisfactory separation between the groups. The permutation test of the PLS-DA model revealed an R^2^ (cum) value of 0.845 concerning the interpretative ability of the model, along with a Q^2^ (cum) value of −0.734 signifying its predictive ability. These values indicated that the model did not overfit and had high predictive accuracy, making it suitable for identification of DEMs (Supplementary Figure S1C). PCA of all samples showed that samples from each group clustered closely together (HFF represented by red dots; AFF represented by blue dots), reflecting effective quality control and ensuring reliability in DEM screening (Supplementary Figure S1D).

### 3.2 Identification and classification of DEMs

**FIGURE 1:**
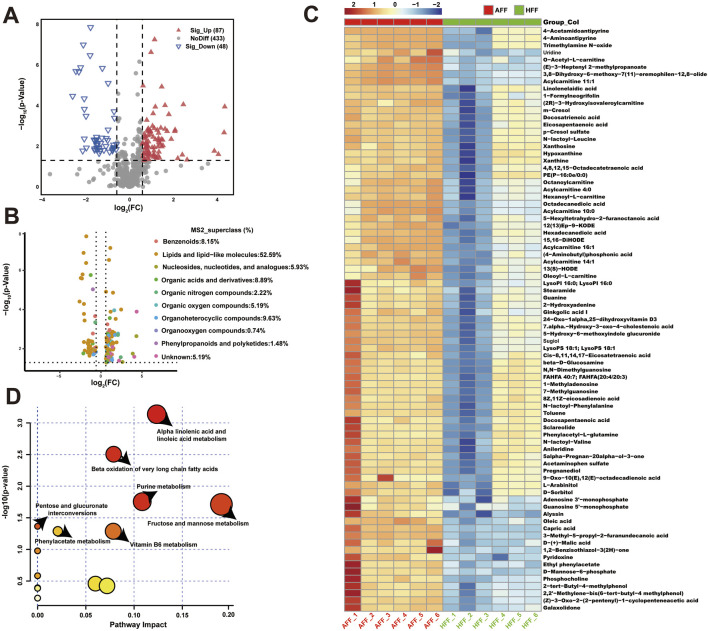
Metabolomic analysis of atretic follicular fluid. **(A)** Volcano plot showing differential expression of metabolites. Red points represent metabolites upregulated in the AFF group, whereas green points indicate downregulated metabolites. **(B)** Volcano plot showing the association of 135 DEMs with MS2 superclass using linear mixed models to compare follicular fluid characteristics between AFF and HFF groups. **(C)** Clustered heatmap of DEM expression between AFF and HFF groups. Each horizontal row represents a DEM, and each column represents a sample. Colors indicate relative expression of DEMs in individual samples. **(D)** Metabolite set enrichment analysis in atretic follicles. Ordinate indicates significance and abscissa denotes pathway impact.

After qualitative and quantitative analysis of the detected metabolites, we identified DEMs between the HFF and AFF groups using the criteria of VIP >1, fold change ≥2 or ≤0.5, and *p* < 0.05. In total, 135 DEMs were identified; 87 showed upregulation and 48 exhibited downregulation (Figure 1A; Supplementary Table S3). Compound classification using the HMDB revealed that DEMs were primarily annotated as “Lipids and lipid-like molecules” (52.59%), “Organoheterocyclic compounds” (9.63%), and “Organic acids and derivatives” (8.89%) (Figure 1B). Additionally, 59 and 76 DEMs were identified in positive and negative ion modes, respectively. Annotation of these DEMs using the MS2class feature in the HMDB database indicated that 11 metabolites (positive ion mode) and 25 metabolites (negative ion mode) were classified as “Fatty acyls,” whereas 21 metabolites (positive ion mode) and 14 metabolites (negative ion mode) were classified as “Glycerophospholipids” (Supplementary Figure S1E).

### 3.3 KEGG enrichment analysis and pathway analysis of DEMs

Most DEMs were enriched in pathways related to glycerophospholipid metabolism, metabolic pathways, biosynthesis of amino acids, purine metabolism, and protein digestion and absorption (Supplementary Figures S1F, G). The findings suggest that these pathways play important roles during follicular development in sheep. To annotate the metabolic pathways and interactions associated with atresia-specific DEMs, we conducted KEGG topological analysis and metabolite–metabolite network analysis of HFF and AFF. Due to the large number of DEMs, 87 metabolites with *p*-values <0.01 were selected for heatmap visualization (Figure 1C). The heatmap revealed that most DEMs were elevated in AFF. KEGG topology analysis identified four key metabolic pathways in follicular fluid during follicular atresia: alpha-linolenic acid and linoleic acid metabolism, beta-oxidation of very long-chain fatty acids, fructose and mannose metabolism, and purine metabolism. These pathways exhibited low *p*-values and high pathway impacts, emphasizing their importance (Figure 1D). Next, we performed metabolite–metabolite association network analysis to investigate the interaction relationships of metabolites specific to atretic follicles. Among the identified metabolites, uridine, guanosine 5′-monophosphate, and O-acetyl-L-carnitine were negatively correlated with other DEMs (Supplementary Figure S1H). We constructed an expression matrix to distinguish the expression patterns of 38 specific metabolites in healthy follicles (Supplementary Figure S2A). KEGG enrichment analysis (Supplementary Figure S2B) combined with joint-pathway analysis (Supplementary Figure S2C) highlighted the importance of glycerophospholipid metabolism, phosphatidylcholine biosynthesis, and ether lipid metabolism in follicular fluid during follicular atresia. Key metabolites, including hippuric acid, malondialdehyde, and phosphocholine, positively regulated the expression of multiple metabolites.

### 3.4 Differences in transcriptomic profiles of granulosa cells during follicular atresia

**FIGURE 2:**
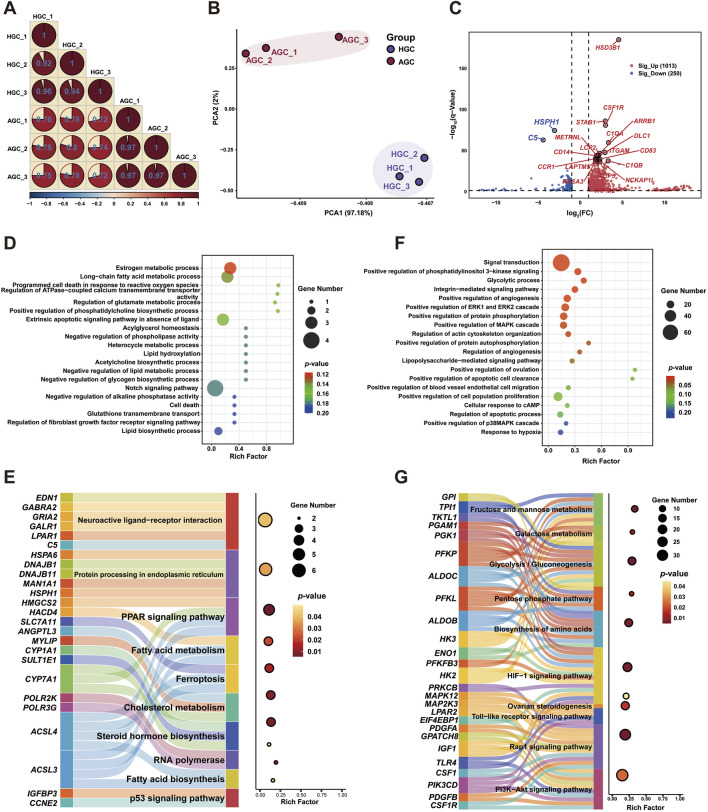
Visualization of global transcriptome profiling of granulosa cells from atretic and healthy follicles **(A)** Correlation analysis of gene expression patterns in each group. **(B)** Principal component analysis of DEGs. **(C)** Volcano plot of DEGs in granulosa cells from atretic and healthy follicles. **(D)** GO enrichment analysis of DEGs from atretic follicles. **(E)** KEGG pathway analysis of DEGs from atretic follicles **(F)** GO enrichment analysis of DEGs from healthy follicles. **(G)** KEGG pathway analysis of DEGs from healthy follicles.

To identify upstream candidate genes linked to the follicular fluid metabolome, transcriptional analysis was conducted on granulosa cell samples corresponding to healthy and atretic follicles. RNA was extracted from three granulosa cell samples per group, and cDNA libraries were constructed. After sequencing and filtering, over 76 million valid reads were obtained. The total mapped rate ranged from 90.42% to 93.82%; all Q20 values exceeded 99.97%, with Q30 values reaching 98.41% (Supplementary Table S4). Additionally, more than 52% of the reads were mapped to exons, and the FPKM density distribution was consistent between healthy and atretic follicle groups (Supplementary Figures S3A, B). Pearson correlation analysis based on gene expression profiles showed that correlations within each group exceeded 0.92, indicating strong similarity among biological replicates and validating the experimental design (Figure 2A). Consistent with this result, PCA revealed distinct distributions between the two groups (Figure 2B). Similar patterns were observed in the heatmap, which highlighted significant differences in gene expression clusters within each sample group (Supplementary Figure S3C).

After sample processing, we performed differential genetic screening to compare atretic and healthy follicles. In total, 1263 DEGs were identified (|log_2_(fold change)| > 1 and false discovery rate <0.05) in granulosa cells from healthy follicles, comprising 1013 upregulated and 250 downregulated genes (Figure 2C; Supplementary Table S5). To further distinguish between upregulated and downregulated DEGs, Gene Ontology biological process analysis was performed separately for these groups. Downregulated DEGs (20%) were highly enriched in functions related to cell metabolism and programmed cell death (Figure 2D). Upregulated DEGs (80%) were predominantly associated with granulosa cell proliferation and follicle development, including processes such as signal transduction, positive regulation of cell population proliferation, and positive regulation of angiogenesis (Figure 2E). KEGG pathway enrichment analysis was performed to evaluate pathways involved in follicle development for both upregulated and downregulated DEGs. Downregulated DEGs were primarily enriched in pathways related to fatty acid metabolism, steroid hormone biosynthesis, ferroptosis, and p53 signaling. Among these, the peroxisome proliferator-activated receptor (PPAR) signaling pathway showed the greatest enrichment (*p* < 0.01), whereas the highest number of genes was enriched in the neuroactive ligand-receptor interaction pathway. Notably, the ferroptosis pathway was significantly enriched in atretic follicles, involving genes such as *SLC7A11* and *ACSL4* (Figure 2F). Additionally, the 1013 upregulated DEGs were significantly enriched (*p* < 0.01) in pathways associated with granulosa cell proliferation and oocyte maturation, including the hypoxia-inducible factor 1 (HIF-1), phosphoinositide 3-kinase (PI3K)–protein kinase B (Akt), and Rap1 signaling pathways, as well as glycolysis/gluconeogenesis (Figure 2G).

To further identify candidate hub genes and their interaction networks among the upregulated and downregulated DEGs, we used the CytoHubba plugin for Cytoscape software. The top 10 hub genes were selected based on maximal clique centrality algorithm scores, as shown in Supplementary Figures S4A, B. The analysis revealed that the most prominent genes in atretic follicles were Toll-like receptor 4 (*TLR4*), integrin subunit beta 2 (*ITGB2*), and interleukin-1 beta (*IL1B*); transforming growth factor beta 2 (*TGFB2*), fibroblast growth factor 7 (*FGF7*), and decorin (*DCN*) were the predominant genes expressed in healthy follicles. Similarly, KEGG pathway enrichment analysis was performed to explore the co-regulatory functions of 20 hub genes. The results highlighted key pathways, including the mitogen-activated protein kinase (MAPK), PI3K–Akt, and Hippo signaling pathways. Atretic follicles were specifically enriched in processes associated with hypoxic stress and programmed cell death, including the nuclear factor (NF)-kappa B and HIF-1 signaling pathways, as well as necroptosis (Supplementary Figure S4C).

### 3.5 Integrated analysis of ferroptosis-related metabolites and genes

**FIGURE 3:**
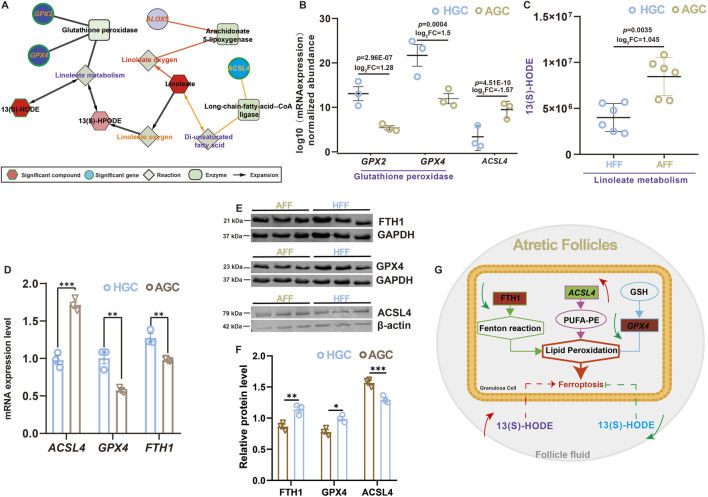
Integrated analysis of ferroptosis-related metabolites and genes **(A)** Connected network of ferroptosis-related genes, enzymes, reactions, and metabolites involved in linoleic acid metabolism. Hexagonal nodes represent compounds, and red nodes were identified in this study. Diamonds represent reactions, squares indicate enzymes, and circles indicate genes; green nodes represent significant findings. **(B)** Expression levels of glutathione peroxidase-related genes according to RNA-seq data. Values are presented as mean ± standard error of the mean (n = 3). **(C)** Scatter plot showing significant alterations in 13(S)-HODE, a key metabolite in linoleic acid metabolism. Values are presented as mean ± standard error of the mean (n = 6). **(D)** Relative expression levels of ferroptosis-related genes in granulosa cells from healthy and atretic follicles, according to qRT-PCR. Values are presented as mean ± standard error of the mean (n = 3). **(E)** Expression of ferroptosis-associated proteins, as determined by Western blot analysis. **(F)** Protein levels of ferroptosis-associated (GPX4, ACSL4, and FTH1) were quantified in atretic follicle and healthy follicle. Expression of GAPDH and β-actin protein were used as an internal control. Values are presented as mean ± standard error of the mean (n = 3). *p < 0.05; **p < 0.01; ***p < 0.001. **(G)** Proposed model illustrating granulosa cell ferroptosis in atretic follicles and the distinct effects of different levels of 13(S)-HODE on follicular fate.

Metabolomics and transcriptomics are powerful omics technologies that provide comprehensive profiles of metabolites and transcripts. To elucidate the interconnected network of ferroptosis-related mRNAs and metabolites in atretic follicles, we utilized the MetScape plugin for Cytoscape to integrate metabolomic and transcriptomic data, linking specific linoleic acid metabolites in follicular fluid with the expression of ferroptosis-related mRNAs in granulosa cell. As shown in Figure 3A, 13(S)-HODE regulates genes involved in the GPX pathway through linoleic acid metabolism, while ACSL4 mediates linoleic acid metabolism through polyunsaturated fatty acids. The analysis revealed that 13(S)-HODE levels were significantly elevated in the follicular fluid of atretic follicles, while *GPX4* mRNA expression decreased and *ACSL4* mRNA expression increased in granulosa cells (Figures 3B,C). These findings suggest that 13(S)-HODE influences granulosa cell ferroptosis and follicle development. We analyzed dynamic changes in four ferroptosis-related genes within atretic follicles (Figure 3D) and verified the RNA-seq results via RT-qPCR (Figure 3E). Western blot analysis demonstrated increased levels of ACSL4 and decreased levels of GPX4 in granulosa cells after follicular atresia (Figures 3E, F). The results indicated a consistent regulatory trend in gene expression, confirming that ferroptosis occurs in atretic follicles (Figure 3G).

### 3.6 13(S)-HODE regulates granulosa cell ferroptosis through GPX4-Mediated lipid peroxidation

**FIGURE 4:**
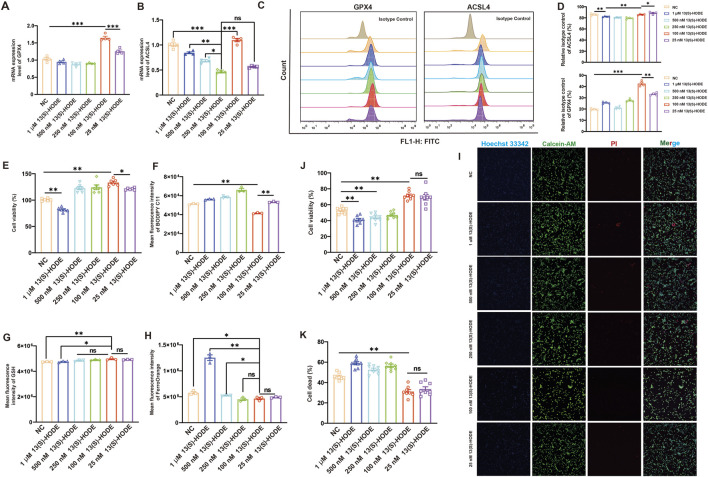
Regulation of ferroptosis in granulosa cells by 13(S)-HODE **(A)** qRT-PCR analysis of relative mRNA expression levels of GPX4 in granulosa cells. **(B)** qRT-PCR analysis of relative mRNA expression levels of ACSL4 in granulosa cells. **(C)** Flow cytometry analysis of GPX4 and ACSL4 (fluorescein isothiocyanate [FITC]) protein expression in granulosa cells treated with various doses of 13(S)-HODE. **(D)** Bar plots showing the mean fluorescence intensity of GPX4 and ACSL4 protein expression. **(E)** Assessment of granulosa cell viability 24 h after 13(S)-HODE treatment using the CCK-8 kit. **(F)** Flow cytometry analysis of mean fluorescence intensity for C11-BODIPY581/591 (lipid peroxidation) in granulosa cells treated with various doses of 13(S)-HODE. **(G)** Granulosa cells were treated as indicated for 24 h, and GSH accumulation was assessed via monochlorobimane staining. **(H)** Flow cytometry analysis of intracellular Fe2+ iron levels via FerroOrange (phycoerythrin fluorescence). **(I)** Fluorescent imaging of living and dead cells in granulosa cells using calcein-AM/PI staining. Nuclei were stained with Hoechst 33342 (blue), living cells were stained with calcein-AM (green), and dead cells were stained with PI (red). Scale bar = 100 μm. **(J)** Histogram showing cell viability based on live cell/total cell ratio using a calcein-AM/Hoechst 33342 double-staining assay. **(K)** Histogram displaying the percentage of dead cells based on dead cell/total cell ratio using a PI/Hoechst 33342 double-staining assay. Values are presented as mean ± standard error of the mean from three independent experiments. Statistical significance of differences among groups was determined using a one-way analysis of variance. *p < 0.05, **p < 0.01, ***p < 0.001.

13(S)-HODE is a lipid signaling molecule involved in lipid metabolism and inflammatory responses. GPX4, a key enzyme that reduces intracellular lipid peroxidation, is essential for preventing ferroptosis; its deletion is sufficient to trigger ferroptosis. To evaluate the direct effect of 13(S)-HODE on GPX4 and its role in granulosa cell ferroptosis resistance, we assessed GPX4 expression levels, cell viability, lipid peroxidation, and Fe^2+^ content in granulosa cells treated with varying concentrations of 13(S)-HODE for 24 h. RT-qPCR analysis of granulosa cells treated with different doses of 13(S)-HODE revealed that *GPX4* expression was significantly upregulated in the 100 nM 13(S)-HODE group compared to other groups (Figure 4A). Protein analysis and quantification using flow cytometry (Forment and Jackson, 2015) confirmed that 100 nM 13(S)-HODE increased GPX4 protein expression, consistent with the transcriptional changes, indicating that 13(S)-HODE serves as a key driver of *GPX4* expression (Figures 4C,D). These findings suggest that low-dose 13(S)-HODE positively regulates GPX4 expression at both mRNA and protein levels. Intriguingly, treatment with 100 nM 13(S)-HODE did not decrease ACSL4 expression at the mRNA or protein level relative to the control group (Figures 4B,D). We speculate that 13(S)-HODE promotes ferroptosis resistance in granulosa cells by enhancing GPX4 expression.

Notably, cells treated with 100 nM 13(S)-HODE for 24 h exhibited higher viability relative to the control group (Figure 4E). Given that *GPX4* is a core gene in the defense against ferroptosis, we analyzed levels of GSH (a crucial substrate for GPX4) and lipid peroxidation products. As shown in Figure 4F, 100 nM 13(S)-HODE treatment significantly suppressed lipid peroxidation compared with the control group. Additionally, GSH levels in granulosa cells were significantly higher in the 100 nM 13(S)-HODE-treated group than in the control group (Figure 4G); intracellular iron ion content was significantly lower in the treated group (Figure 4H). These findings indicate that low-dose 13(S)-HODE mitigates intracellular lipid peroxidation by increasing GSH levels, thus inhibiting ferroptosis. Interestingly, treatment with high doses of 13(S)-HODE (500 nM or 1 μM) induced cell death, which was accompanied by a significant increase in Fe^2+^ content (Figure 4I). Live/dead cell imaging of granulosa cells was conducted using a calcein-AM/PI kit. Calcein-AM hydrolyzed into calcein in living cells emits green fluorescence; PI selectively binds to DNA in dead cells, producing red fluorescence. Cell viability rates were calculated using ImageJ software. Quantitative analysis revealed survival rates of 58.82%, 71.5%, and 70.1% in the 0, 100 nM, and 25 nM groups, respectively (Figure 4J). Strikingly, more than 50% of granulosa cells died after treatment with 500 nM or 1 μM 13(S)-HODE for 24 h, as evidenced by live/dead cell staining with calcein and PI fluorescence dyes (Figure 4K). These results collectively confirmed that low-dose 13(S)-HODE significantly enhances granulosa cell resistance to ferroptosis. However, under 1 μM 13(S)-HODE conditions, Fe^2+^-induced sensitivity to ferroptosis prevents GPX4-mediated resistance.

### 3.7 13(S)-HODE alleviates RAS-Selective lethal 3 (RSL3)-Induced ferroptosis in granulosa cells

**FIGURE 5:**
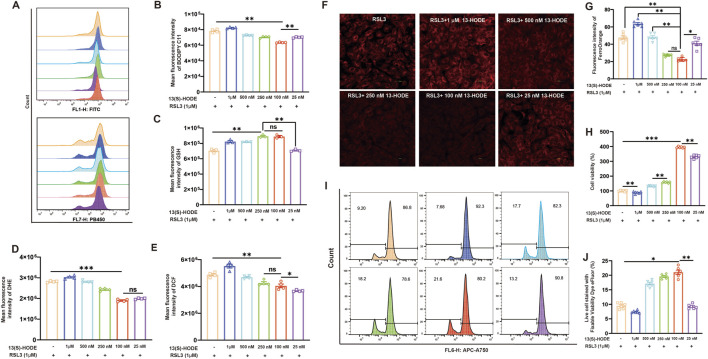
13(S)-HODE inhibited ferroptosis in RSL3-treated granulosa cells. **(A)** Original histograms of BODIPY 581/591 C11 and monochlorobimane fluorescence, measured by flow cytometry. **(B)** Bar plots of lipid peroxidation levels in granulosa cells exposed to RSL3 and 13(S)-HODE. **(C)** Bar plots of GSH levels in granulosa cells treated with or without RSL3 and 13(S)-HODE. **(D)** Flow cytometry measurements of dihydroethidium (DHE) fluorescence corresponding to cellular ROS levels. Bar plots represent data from six experiments. **(E)** Flow cytometry analysis using dichlorodihydrofluorescein diacetate (DCFH-DA) staining to measure ROS production in cultured granulosa cells. ROS production was assessed based on changes in the fluorescence intensity of dichlorodihydrofluorescein (DCF; oxidation product of DCFH-DA). **(F)** Fluorescence images of FerroOrange staining in granulosa cells. Red fluorescence intensity represents Fe2+ level. **(G)** Quantification of fluorescence intensity from FerroOrange staining. **(H)** Bar plots showing cell viability measured by CCK-8 after 24 h of exposure to 0, 1 μM, 500 nM, 250 nM, 100 nM, or 25 nM 13(S)-HODE during RSL3 treatment. **(I)** Flow cytometry analysis of live/dead cell populations using fixable viability dyes. Population profiles are displayed in the viability dye histogram. **(J)** Positive eFluor 780 labeling distinguishing live cells (left) from dead cells (right). Values are presented as mean ± standard error of the mean from three independent experiments. Statistical significance of differences among groups was determined using a one-way analysis of variance. *p < 0.05, **p < 0.01, ***p < 0.001.

RSL3 is a potent ferroptosis inducer that inhibits GPX4, resulting in lipid peroxide accumulation and oxidative damage that ultimately triggers ferroptosis in susceptible cells (Zhou Q. et al., 2024). To investigate the protective role of 13(S)-HODE against ferroptosis in granulosa cells, we utilized RSL3 (1 μM, 24 h) to induce cellular ferroptosis. Flow cytometry analysis revealed that levels of lipid peroxidation and ROS were significantly elevated in the RSL3-treated group. However, treatment with low concentrations of 13(S)-HODE significantly reduced the fluorescence intensity of lipid peroxidation (Figures 5A,B). Furthermore, GSH levels decreased in the RSL3 group but were restored after treatment with 100 nM 13(S)-HODE (Figure 5C), while ROS accumulation was significantly reduced, as shown in Figures 5D,E. The effect of 13(S)-HODE on ferroptosis was further explored by measuring Fe^2+^ levels using the fluorescent probe FerroOrange. Stimulation of granulosa cells with RSL3 led to the accumulation of intracellular Fe^2+^, as indicated by increased red fluorescence intensity. However, 13(S)-HODE treatment at low concentrations reduced this fluorescence intensity (Figures 5F,G). RSL3-induced ferroptotic cell death was observed, but treatment with 100 nM 13(S)-HODE reversed this effect, significantly improving cell survival (Figure 5H). Cell viability analysis demonstrated a survival rate of 9.20% in cells treated with RSL3 alone, which increased to 21.60% in cells exposed to 100 nM 13(S)-HODE combined with RSL3 (Figures 5I,J). These findings confirm that granulosa cells exhibit stronger resistance to ferroptosis in the presence of a low concentration of 13(S)-HODE. This protective effect against ferroptosis suggests that 13(S)-HODE plays a crucial role in preventing follicular atresia.

### 3.8 High-dose 13(S)-HODE promotes iron metabolism-mediated ferroptosis by upregulating TFRC and FTH1

**FIGURE 6:**
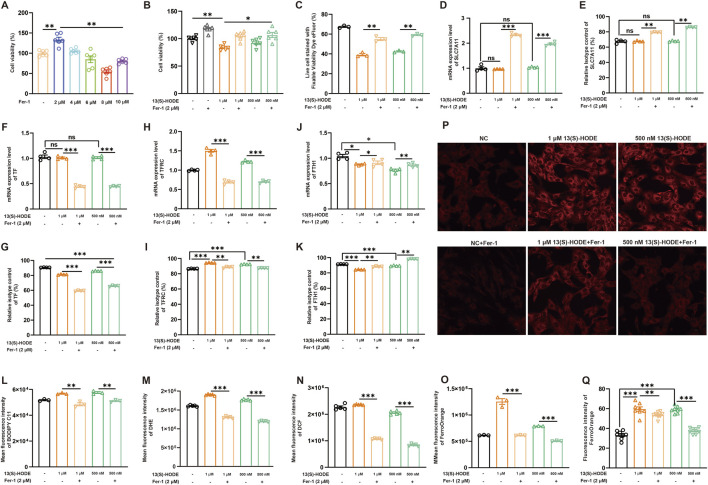
High-dose 13(S)-HODE promotes iron metabolism-mediated ferroptosis in granulosa cells **(A)** Granulosa cells were treated with five different concentrations of the ferroptosis inhibitor ferrostatin-1 (Fer-1) for 24 h, and cell viability was measured using the CCK-8 assay. **(B)** Granulosa cells were treated with Fer-1 (2.0 μM), 1 μM 13(S)-HODE, 1 μM 13(S)-HODE + Fer-1 (2.0 μM), 500 nM 13(S)-HODE, and 500 nM 13(S)-HODE + Fer-1 (2.0 μM); cell viability was measured using the CCK-8 assay. **(C)** Flow cytometry analysis of live/dead cell populations using fixable viability dyes. Population profiles are displayed in the viability dye histogram. **(D,F,H,J)** RT-qPCR analysis of relative mRNA expression levels of ferroptotic genes (SLC7A11, TF, TFRC, and FTH1) in granulosa cells treated with high doses of 13(S)-HODE during Fer-1 treatment (n = 4). β-actin served as the reference gene. **(E,G,I,K)** Flow cytometry analysis of SLC7A11, TF, TFRC, and FTH1 protein expression levels in granulosa cells treated with high doses of 13(S)-HODE during Fer-1 treatment. **(L)** Flow cytometry measurements of lipid peroxidation levels in granulosa cells. Bar plots represent data from six experiments. **(M)** Flow cytometry measurements of DHE fluorescence corresponding to cellular ROS levels. Bar plots represent data from six experiments. **(N)** Intracellular ROS content detected using fluorescent DCFH-DA probes. Bar plots represent data from six experiments. **(O)** Fluorescence intensity analysis of intracellular Fe2+ content in granulosa cells stained with FerroOrange. Bar plots represent data from six experiments. **(P,Q)** Fluorescence images of FerroOrange staining in granulosa cells. Red fluorescence intensity represents Fe2+ level. Bar plots represent data from six experiments.

To confirm the high-dose 13(S)-HODE-mediated regulation of ferroptosis in granulosa cells, we explored the effects of ferrostatin-1 (Fer-1), a ferroptosis inhibitor. The effect of 2 µM Fer-1 on cell viability is shown in Figure 6A; this served as a positive control. Subsequently, we assessed cell viability and cell death. As shown in Figures 6B,C, treatment with 1 μM or 500 nM 13(S)-HODE induced cell death, which was rescued by Fer-1 treatment. These findings suggest that 13(S)-HODE-induced cell death occurs via ferroptosis. To investigate the mechanisms underlying high-dose 13(S)-HODE-induced ferroptosis, we analyzed two main systems involved in ferroptosis: System Xc^−^ and iron metabolism. System Xc^−^ consists of the SLC7A11 (xCT) and SLC3A2 subunits. The results indicated that 13(S)-HODE treatment did not alter SLC7A11 expression in granulosa cells (Figures 6D,E). Next, we examined changes in iron metabolism-related genes at both the mRNA and protein levels, including transferrin (iron transport), TFRC (iron uptake), and FTH1 (iron storage). High doses of 13(S)-HODE significantly increased the protein levels of these genes (Figures 6F,G). Notably, as shown in Figures 6H–K, TFRC was significantly upregulated, whereas FTH1 was significantly downregulated in response to treatment with 1 μM or 500 nM 13(S)-HODE; these effects were reversed by Fer-1 treatment. The results suggest that high-dose 13(S)-HODE promotes intracellular Fe^3+^ accumulation in granulosa cells, likely through increased TFRC expression and reduced FTH1 expression. Considering that ferroptosis involves iron-dependent lipid peroxidation, we evaluated levels of lipid peroxidation and ROS. Treatment with 1 μM or 500 nM 13(S)-HODE significantly increased lipid peroxidation and ROS accumulation in granulosa cells. However, when cells were treated with a combination of Fer-1 and 13(S)-HODE, these increases were substantially attenuated (Figures 6L–N). Fluorescence staining and flow cytometry confirmed that 13(S)-HODE induced the accumulation of Fe^2+^ (Figures 6O–Q). Notably, Fer-1 treatment significantly alleviated intracellular iron accumulation caused by high-dose 13(S)-HODE. In summary, these findings collectively suggest that high-dose 13(S)-HODE induces ferroptosis through a TFRC/FTH1-mediated mechanism involving Fe^2+^ overload in granulosa cells.

## 4 Discussion

The death of granulosa cells inhibits follicle development and promotes follicular atresia. Although granulosa cell apoptosis plays an important role in follicular atresia, increasing evidence suggests that additional cell death pathways are involved in its regulation. Ferroptosis, a recently discovered form of regulated cell death driven by iron-dependent lipid peroxidation, has received limited attention in the context of follicular atresia. In this study, we conducted a comprehensive analysis of the granulosa cell transcriptome and follicular fluid metabolome in healthy and atretic follicles. Our findings highlight the role of ferroptosis in the progression of atretic follicles and demonstrate that 13(S)-HODE, a specific metabolite closely associated with atretic follicles, regulates the molecular mechanisms that underlie ferroptosis in granulosa cells.

Follicular fluid provides a specialized microenvironment for follicle development. The present study showed that 87 metabolites were upregulated in the follicular fluid of atretic follicles, most of which were lipids closely associated with follicle development and oocyte quality. Previous metabolomic studies have indicated that follicular dysfunction is closely linked to lipid metabolism disorders (Liang et al., 2021; Hwang et al., 2022). Purine metabolism influences a wide range of cellular processes, including nucleic acid synthesis, energy production, DNA/RNA synthesis, and biological signaling (Huang et al., 2021). It also serves as a source of genetic material for normal cell function (Unnikrishnan et al., 2019). In this study, we observed xanthine and hypoxanthine upregulation in the follicular fluid of atretic follicles, consistent with previous findings that purine metabolism is disrupted in patients with ovarian dysfunction (Wei et al., 2023). Furthermore, some studies have indicated that decreased oocyte quality may be associated with increased purine metabolism (Wen et al., 2010). Polyunsaturated fatty acids are key components of follicular fluid; linoleic acid, the most abundant polyunsaturated fatty acid, exhibits a negative effect on bovine oocyte development (Marei et al., 2010). High levels of linoleic acid can increase neutral lipid accumulation. Zhang and Wu (2024) reported significantly elevated linoleic acid levels in patients with polycystic ovary syndrome; they also demonstrated that linoleic acid induces granulosa cell apoptosis and ovarian inflammation. Fatty acids containing more than 18 carbons are considered very long-chain fatty acids. Among these, beta-oxidation is the primary pathway for fatty acid degradation and contributes approximately 60%–70% of energy generation (Houten et al., 2016). Studies have also shown that fatty acid oxidation reduces the intracellular levels of free non-esterified fatty acids (Oyanagi et al., 2011). Our findings indicate that beta-oxidation is increased in atretic follicles, potentially mitigating lipotoxicity and fatty acid peroxidation. In summary, follicular atresia may be linked to the upregulation of purine metabolism and disruptions in lipid metabolism.

Oocyte quality partially depends on the follicular microenvironment. Our study revealed that 48 metabolites were significantly upregulated in healthy follicles, with particular enrichment in pathways related to ether lipid metabolism, phosphatidylcholine biosynthesis, and glycerophospholipid metabolism. There is evidence that ether lipids are positively correlated with oocyte quality and competence; they may also contribute to granulosa cell ferroptosis as substrates for phospholipid peroxidation (Zhang et al., 2024). These findings highlight the need for further investigation concerning the roles of lipid peroxidation stress and ferroptosis in follicular atresia. Glycerophospholipids are essential structural components of biological membranes and precursors for many signaling molecules. Previous studies have demonstrated that reduced glycerophospholipid levels are strongly correlated with lower fertilization rates (Liu et al., 2019). Phosphatidylcholine has also been identified as a key factor influencing embryo quality (Wang et al., 2021). Pathway analysis showed that these metabolites are clustered together, leading to the hypothesis that lipids and glycerophospholipid species may serve as biomarkers for follicular developmental competence and oocyte quality. In summary, alterations in lipid metabolism are closely linked to follicle development.

Alterations in key genes strongly influence pathway activities because persistent gene signals are primary drivers of abnormal pathway function (Liu et al., 2015). The present study showed that ferroptosis and p53 signaling pathways were significantly enriched in atretic follicles; the PPARγ signaling pathway exhibited the greatest enrichment. Previous studies have revealed increased PPARγ expression in granulosa cell tumors (Yu et al., 2024). These findings emphasize that abnormal lipid metabolism is a key factor in follicular atresia. p53 plays critical roles in regulating apoptosis, cell cycle arrest, inflammation, and autophagy (Zanjirband and Rahgozar, 2019). Additionally, 1013 DEGs highly expressed in healthy follicles were significantly enriched in pathways associated with granulosa cell proliferation and oocyte maturation. The PI3K–Akt pathway is a pivotal signaling cascade required for follicle-stimulating hormone signal transduction (Hunzicker-Dunn et al., 2012). Furthermore, the HIF-1α pathway regulates anaerobic metabolism by controlling the metabolic switch to glycolysis in granulosa cells (Zhou et al., 2018). The glycolytic pathway in granulosa cells is needed to provide the energy required for follicle maturation and development (Kansaku et al., 2017). Combined with the metabolomic findings from HFF, this study demonstrated that fatty acids and their derivatives regulate follicle development by participating in energy metabolism, steroid hormone synthesis, and signal transduction.

To elucidate the mechanisms underlying follicular atresia and explore potential diagnostic biomarkers to improve follicular development, we identified candidate biomarkers in atretic follicles, including *TLR4*, *ITGB2*, and *IL1B*, through protein-protein interaction network analysis using the CytoHubba plugin in Cytoscape. Toll-like receptors constitute a family of 10 cellular receptors responsible for detecting pathogens and initiating innate immune responses (Miller et al., 2005). A previous study demonstrated that bovine granulosa cells initiate an innate immune response via TLR4-dependent inflammation, leading to disrupted meiotic competence (Bromfield and Sheldon, 2011). IL1B is a key pro-inflammatory mediator; the promoter region of *ITGB2* is an important regulator of follicle development and oocyte maturation (Guo et al., 2018). The present results are consistent with previous findings, where the ITGB2 expression level was higher in atretic follicles than in healthy follicles (Kisliouk et al., 2007). These observations suggest that *TLR4* and *ITGB2* can serve as key markers for identifying follicular atresia. In healthy follicles, hub genes associated with maintaining folliculogenesis and follicle survival were identified. Among these, *DCN* regulates granulosa cell apoptosis and the cell cycle (Peng et al., 2016), *TGFB2* plays a role in cumulus expansion (Hao et al., 2022), and *FGF7* may be an essential regulator of follicle survival and oocyte growth (Zhang Y. et al., 2023). Transcriptomic and metabolomic studies provide critical insights into follicular atresia at the gene and metabolite levels, respectively. The integration of transcriptomic and metabolomic data revealed that linoleic acid metabolism is closely associated with follicular atresia; 13(S)-HODE regulates granulosa cell ferroptosis by inducing GPX activity. There is increasing evidence that iron and lipid peroxide accumulation contribute to ovarian insufficiency (Du et al., 2023). Our results also indicate the presence of lipid metabolism disorders and phospholipid peroxidation in atretic follicles. Therefore, we focused on investigating the mechanism by which 13(S)-HODE influences granulosa cell ferroptosis.

13(S)-HODE is produced through the oxidation of linoleic acid, a process that can occur in various cell types (Fang et al., 1999). This molecule can alter the properties of phosphatidylcholine to directly affect gene transcription (Cho and Ziboh, 1994). Previous studies have shown that 13(S)-HODE, a lipoxygenase metabolite of linoleic acid, regulates cellular processes by inhibiting proliferation, inducing apoptosis, and increasing oxidative stress (Hampel et al., 2006). There is evidence that 13(S)-HODE selectively inhibits membrane-bound protein kinase C-beta activity in hyperproliferative skin (Mani et al., 1998). Additionally, elevated 13(S)-HODE levels have been reported in the plasma of patients with nonalcoholic steatohepatitis (Feldstein et al., 2010), suggesting a role in mitigating oxidative stress. However, the effect of 13(S)-HODE on ferroptosis remains underexplored. In this study, we investigated the therapeutic effects of 13(S)-HODE on ferroptosis and elucidated its mechanism of action in granulosa cells. Our findings confirmed that, compared with the NC group, low-dose 13(S)-HODE treatment (100 nM and 25 nM) enhanced mRNA and protein expression levels of GPX4 and significantly reduced ACSL4 expression in granulosa cells. These results indicate that low doses of 13(S)-HODE mitigate lipid peroxidation and iron accumulation. Collectively, our findings strongly suggest that 13(S)-HODE can serve as a therapeutic agent for ferroptosis. To further examine the effect of 13(S)-HODE on ferroptosis, we utilized RSL3 to induce ferroptosis in granulosa cells. Our results confirmed that 13(S)-HODE treatment increased the mRNA and protein levels of GPX4 while decreasing ACSL4 expression compared with the RSL3-treated group. We also investigated the effects of 13(S)-HODE on iron metabolism and lipid peroxidation during RSL3 treatment. We found that 100 nM 13(S)-HODE treatment reduced Fe^2+^ levels and enhanced GSH activity in cells treated with ferroptosis inducers. Additionally, flow cytometry showed that 100 nM 13(S)-HODE treatment inhibited lipid peroxidation. Taken together, these findings indicate that ferroptosis causes granulosa cell death and that low doses of 13(S)-HODE exert an inhibitory effect on this process. Intriguingly, high doses of 13(S)-HODE increased granulosa cell iron overload. Excessive free iron accelerates ROS production via the Fenton reaction, exacerbating ferroptosis (Ru et al., 2024). Therefore, it is important to determine whether high doses of 13(S)-HODE induce ferroptosis in granulosa cells by altering iron homeostasis. Our study revealed that the ferroptosis inhibitor Fer-1 reversed the increases in iron ion levels and ROS production caused by high doses (1 μM and 500 nM) of 13(S)-HODE. As a regulator of iron uptake, TFRC rigorously controls intracellular iron levels (Torti et al., 2018). Here, we found that elevated TFRC expression increased iron ion entry, thereby enhancing granulosa cell susceptibility to ferroptosis. FTH1, an intracellular protein essential for maintaining iron homeostasis, binds Fe^2+^ and stores it in a stable form (Deng et al., 2024). In the present study, 1 μM 13(S)-HODE treatment caused substantial downregulation of FTH1 expression, which was reversed by Fer-1 treatment. These findings strongly imply that 13(S)-HODE increases ferroptosis sensitivity by upregulating TFRC and downregulating FTH1. Finally, our findings suggest that 13(S)-HODE’s biphasic effects has clinical relevance. 13(S)-HODE, as a key oxidative product in linoleic acid metabolism, can enhance ovarian follicle development by reducing reactive oxygen species and lipid peroxidation in granulosa cell. With excessive intake of linoleic acid, the high levels of 13(S)-HODE produced may induce granulosa cell oxidative stress and metabolic disorders, leading to follicular atresia. Moving forward, linoleic acid metabolism and ferroptosis in GCs are essential for ovarian recovery in patients with premature ovarian insufficiency (POI), and promoting effects of 13(S)-HODE on follicular development ability should be considered. In addition, our findings that measurements of lipid peroxidation, ROS, and Fe^2+^, in addition to apoptosis, in the follicle should also be considered in the assessment of follicular atresia. In conclusion, this study provides a new perspective for reproductive medicine to intervene in follicular development from the perspective of lipid metabolism regulation, which suggests that follicle atresia is closely related to oxidative stress caused by abnormal linoleic acid metabolism, and it will help to clarify the mechanism of action of abnormal linoleic acid in PCOS patients and promote the development of precision therapy for PCOS. Meanwhile, the biological studies targeting 13(S)-HODE may become a new strategy for the treatment of ferroptosis and be used to improve oocyte quality improvement in the assisted reproductive technology.

## 5 Conclusion

**FIGURE 7:**
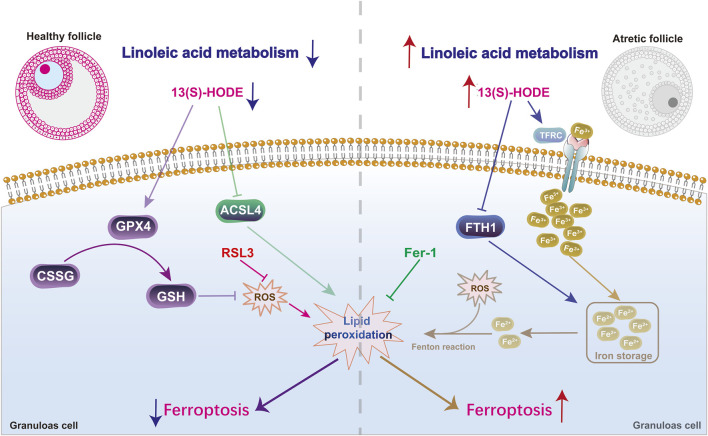
13(S)-HODE drives granulosa cell ferroptosis via the linoleic acid metabolism pathway. At low levels, 13(S)-HODE alleviates follicular atresia by targeting GPX4 and reducing ferroptosis-mediated cell death. However, high doses of 13(S)-HODE induce ferroptosis in granulosa cells through the TFRC/FTH1-mediated iron metabolism pathway.

This integrated multi-omics study demonstrated that ferroptosis occurs in atretic follicles. The findings indicate that 13(S)-HODE may regulate granulosa cell ferroptosis, although this effect is not dose-dependent (Figure 7). Specifically, low doses of 13(S)-HODE alleviate follicular atresia by targeting GPX4 and reducing ferroptosis-mediated cell death, whereas high doses induce ferroptosis in granulosa cells by increasing iron uptake and accumulation. Overall, this work suggests that inhibition of ferroptosis might be a feasible strategy to ameliorate the follicular development of female animals, and highlights the potential for linoleic acid metabolite 13(S)-HODE to serve as protective agents during reproductive outcomes.

## Data Availability

The datasets presented in the study are deposited in the Sequence Read Archive (SRA) repository, accession number PRJNA1240767. This data can be found here: www.ncbi.nlm.nih.gov/bioproject/PRJNA1240767.
