# Supplementary material for: Linoleic acid metabolite 13-Hydroxyoctadecadienoic acid as a biphasic ferroptosis modulator in granulosa cells: multi-omics analysis of ovine atretic follicles
Source: Front Cell Dev Biol. 2025 May 30;13:1610621. doi: 10.3389/fcell.2025.1610621 (PMC12162603; doi:10.3389/fcell.2025.1610621)
Supplement: Supplementary file 1 [file Presentation1.pdf]

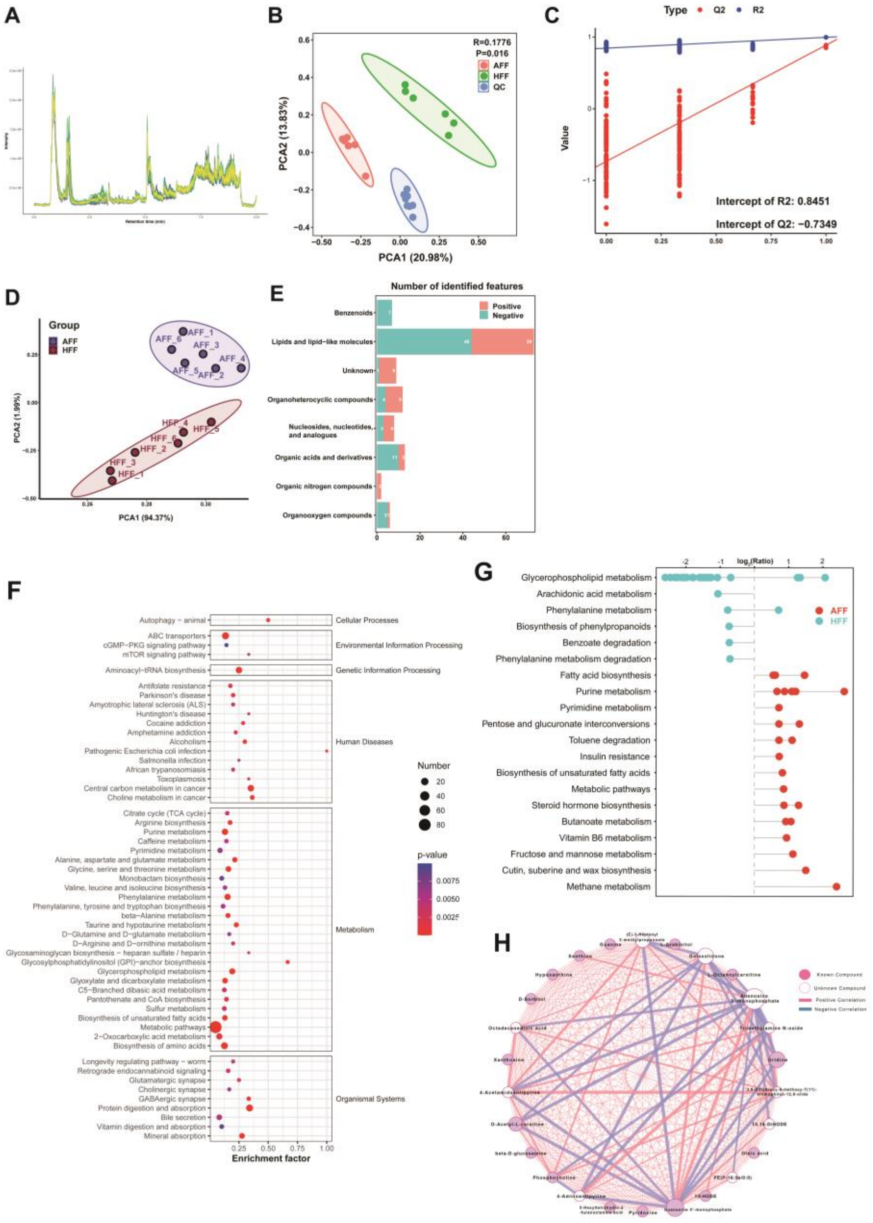

Figure S1. Multivariate statistical analysis of metabolomics data

(A) Total ion current plots of metabolites.

(B) PCA plots and three-dimensional visualizations of differential groupings and quality control (QC).

(C) Two-dimensional score plot by OPLS-DA of follicular fluid samples from the healthy follicle group (red) and atretic follicle group (dark blue). In terms of Q2, the model is not considered overfitted if the y-axis value does not exceed 0.05. When Q2 for the actual model (far right) and Q2 for the random label model are close, overfitting is suspected.

(D) Clustering analysis of metabolite data from AFF and HFF groups.

(E) Classification of DEMs between granulosa cells from atretic and healthy follicles.

(F) Identification and enrichment analysis of DEMs.

(G) KEGG pathway annotation of DEGs. The number in each spot represents the number of proteins.

(G) Metabolite–metabolite interaction network analysis of annotated DEMs in atretic follicles. Negative associations are depicted with blue lines, and positive associations are depicted with red lines

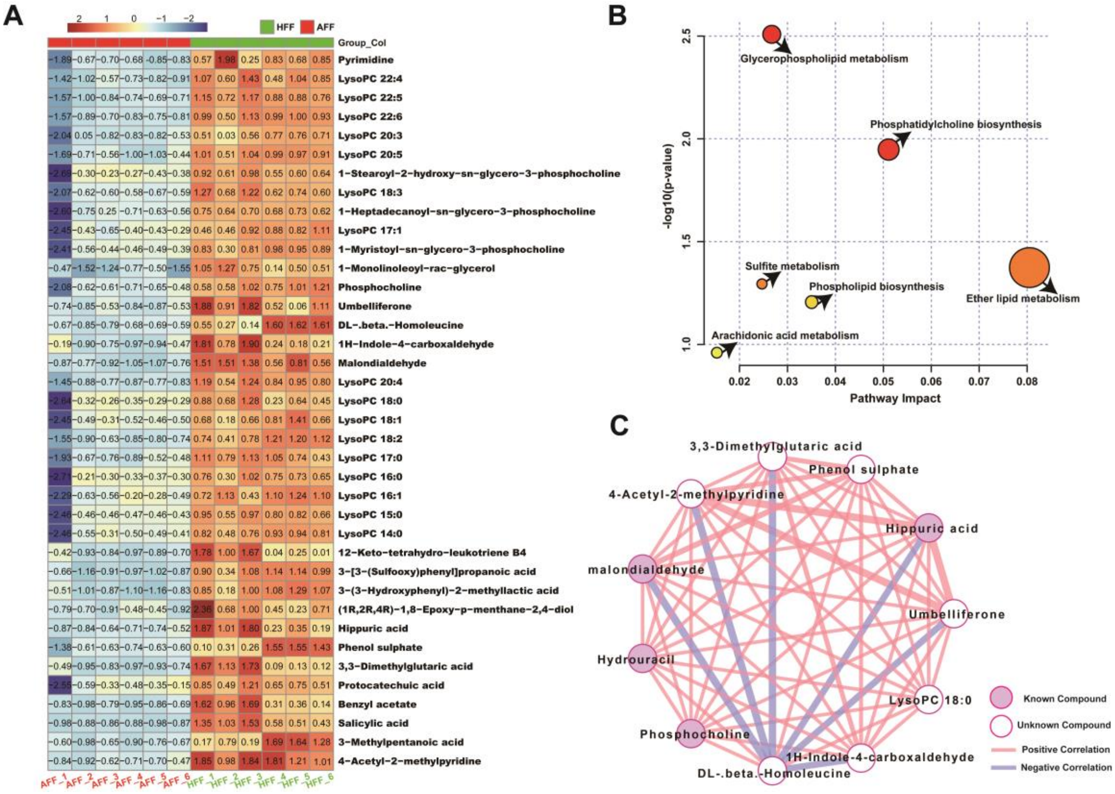

Figure S2. Metabolomic analysis of healthy follicular fluid.

(A) Clustered heatmap of DEM expression between AFF and HFF groups. Each horizontal row represents a DEM, and each column represents a sample. Colors indicate relative expression of DEMs in individual samples.

(B) Metabolite set enrichment analysis in healthy follicles. Ordinate indicates significance and abscissa denotes pathway impact.

(C) Metabolite–metabolite interaction network analysis of annotated DEMs in healthy follicles. Negative associations are depicted with blue lines, and positive associations are depicted with red lines.

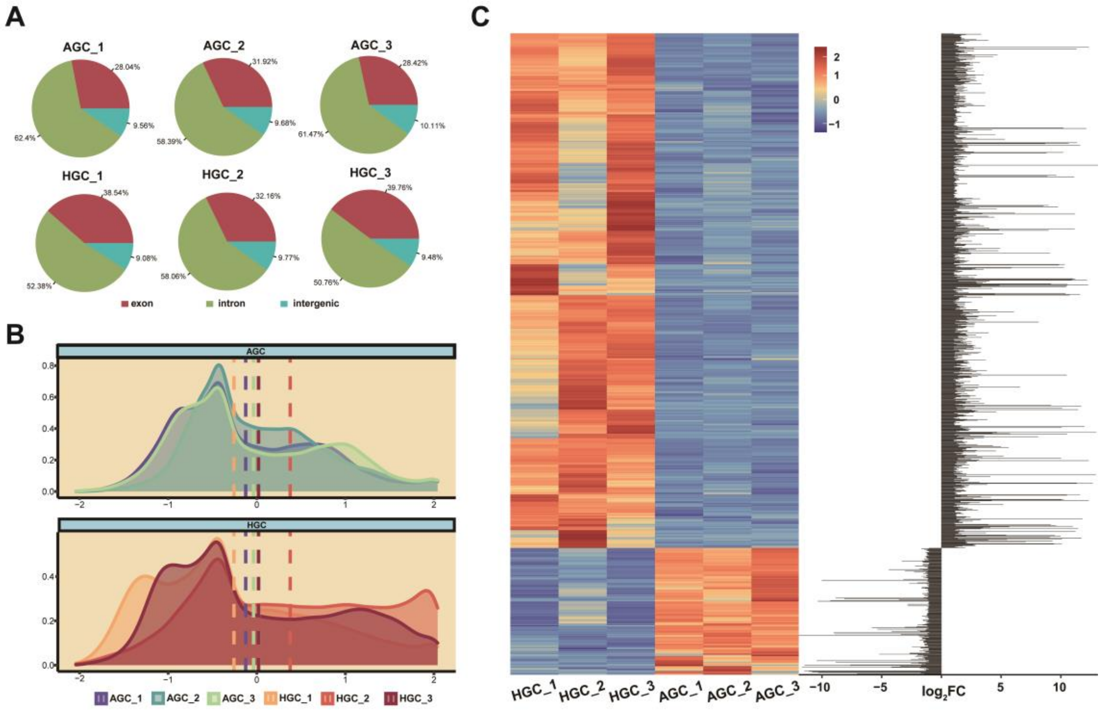

Figure S3. Overview of transcriptome sequencing data.

(A) Relative expression density of transcripts.

(B) Relative expression levels of transcripts.

(C) Heatmap of DEGs.

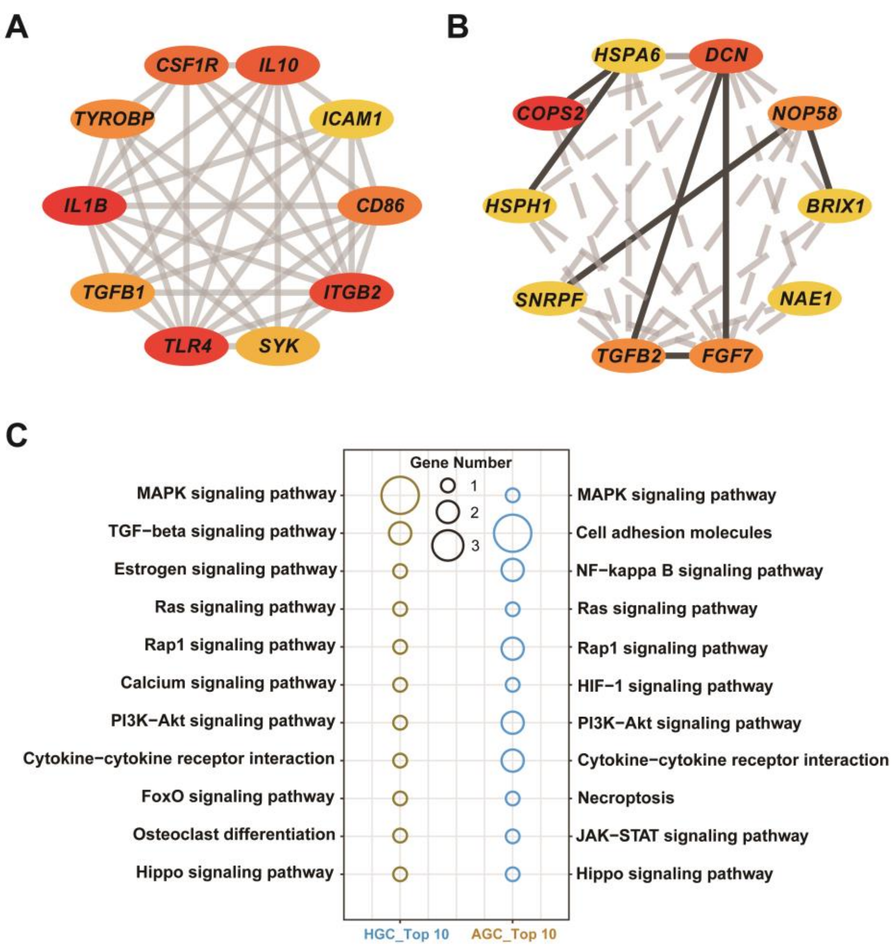

Figure S4. Visualization of hub gene networks using the maximal clique centrality (MCC) algorithm

(A) PPI network of the top 10 hub genes in granulosa cells from atretic follicles. Edges represent connections between proteins, whereas nodes represent genes. Red nodes indicate genes with the highest MCC scores, and yellow nodes indicate genes with the lowest MCC scores.

(B) PPI network of the top 10 hub genes in granulosa cells from healthy follicles. Edges represent connections between proteins, whereas nodes represent genes. Red nodes indicate genes with the highest MCC scores, and yellow nodes indicate genes with the lowest MCC scores.

(C) Representative KEGG results for the top 10 hub genes in granulosa cells from healthy and atretic follicles.

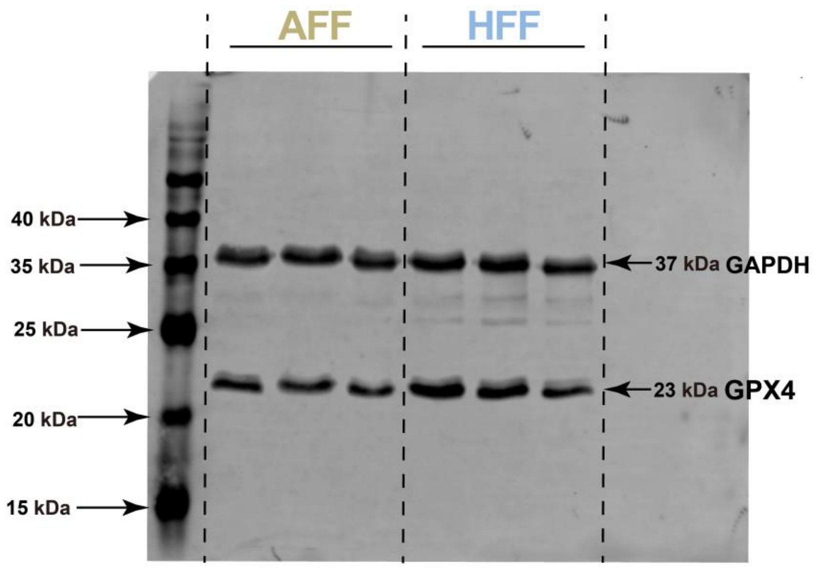
AFF
HFF
40 kDa
35 kDa
25 kDa
20 kDa
15 kDa
37 kDa GAPDH
23 kDa GPX4

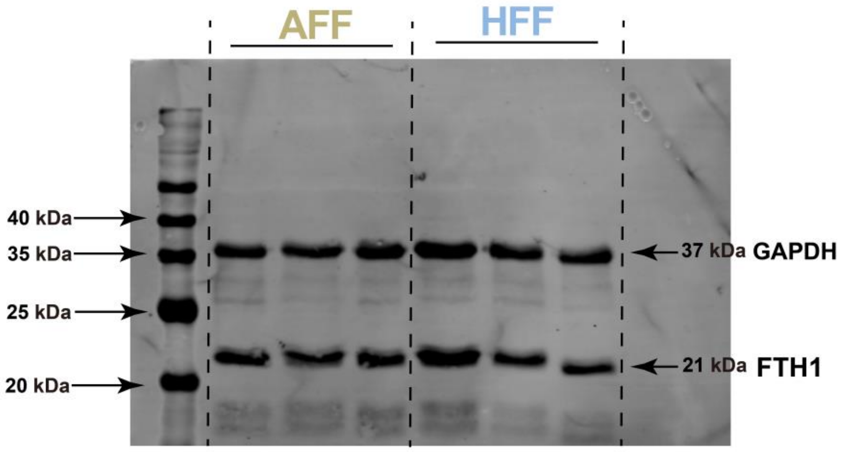
AFF
HFF
40 kDa
35 kDa
25 kDa
20 kDa
37 kDa GAPDH
21 kDa FTH1

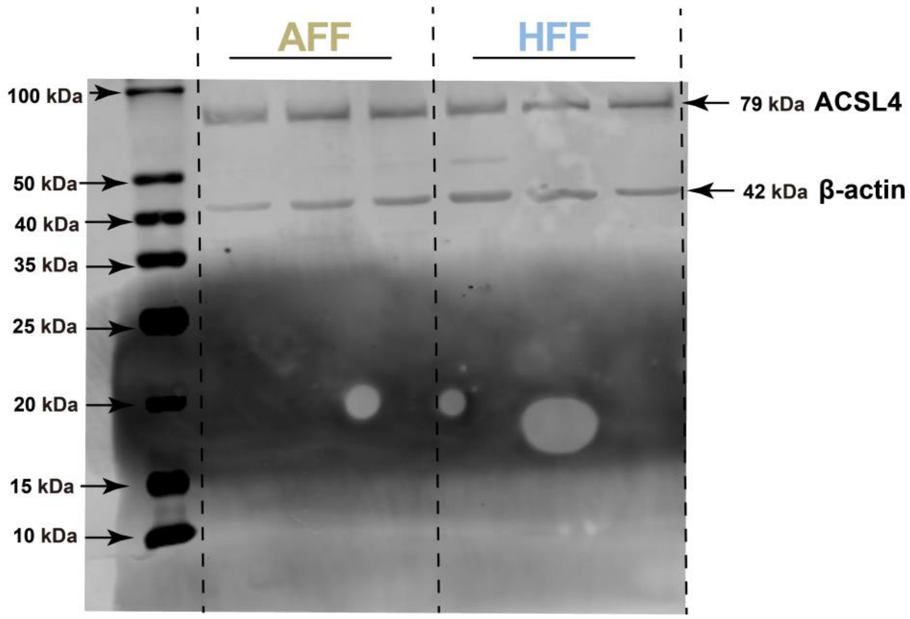
AFF
HFF
100 kDa
50 kDa
40 kDa
35 kDa
25 kDa
20 kDa
15 kDa
10 kDa
79 kDa ACSL4
42 kDa β-actin
